# Hybrid weakness in a rice interspecific hybrid is nitrogen-dependent, and accompanied by changes in gene expression at both total transcript level and parental allele partitioning

**DOI:** 10.1371/journal.pone.0172919

**Published:** 2017-03-01

**Authors:** Shuai Sun, Ying Wu, Xiuyun Lin, Jie Wang, Jiamiao Yu, Yue Sun, Yiling Miao, Qiuping Li, Karen A. Sanguinet, Bao Liu

**Affiliations:** 1 Key Laboratory of Molecular Epigenetics of the Ministry of Education (MOE), Northeast Normal University, Changchun China; 2 Department of Crop and Soil Sciences, Washington State University, Pullman, Washington, United States of America; 3 Jilin Academy of Agricultural Sciences, Changchun China; China National Rice Research Institute, CHINA

## Abstract

**Background:**

Hybrid weakness, a phenomenon opposite to heterosis, refers to inferior growth and development in a hybrid relative to its pure-line parents. Little attention has been paid to the phenomenological or mechanistic aspect of hybrid weakness, probably due to its rare occurrence.

**Methodology/Principal findings:**

Here, using a set of interspecific triploid F1 hybrids between *Oryza sativa*, ssp. *japonica* (genome AA) and a tetraploid wild rice species, *O*. *alta* (genome, CCDD), we investigated the phenotypic and physiological differences between the F1 hybrids and their parents under normal and nitrogen-limiting conditions. We quantified the expression levels of 21 key genes involved in three important pathways pertinent to the assayed phenotypic and physiological traits by real-time qRT-PCR. Further, we assayed expression partitioning of parental alleles for eight genes in the F1 hybrids relative to the *in silico* “hybrids” (parental cDNA mixture) under both normal and N-limiting conditions by using locus-specific cDNA pyrosequencing.

**Conclusions/Significance:**

We report that the F1 hybrids showed weakness in several phenotypic traits at the final seedling-stage compared with their corresponding mid-parent values (MPVs). Nine of the 21 studied genes showed contrasted expression levels between hybrids and parents (MPVs) under normal vs. N-limiting conditions. Interestingly, under N-limiting conditions, the overtly enhanced partitioning of maternal allele expression in the hybrids for eight assayed genes echo their attenuated hybrid weakness in phenotypes, an observation further bolstered by more resemblance of hybrids to the maternal parent under N-limiting conditions compared to normal conditions in a suite of measured physiological traits. Our observations suggest that both overall expression level and differential partitioning of parental alleles of critical genes contribute to condition-specific hybrid weakness.

## Introduction

Hybrid vigor or heterosis refers to the phenomenon of superior performance in an F1 hybrid compared to its inbred parents and has been a focus of study owning to its significance in crop production [1]. Heterosis has been recognized as a general phenomenon [2–4] and has been applied widely to maximize production in many important crops such as maize, rice and wheat [5–7]. In contrast, hybrid weakness, which is the opposite of heterosis, is understudied likely due to its uncommon occurrence. Nevertheless, understanding the molecular underpinnings of hybrid weakness may shed novel insights into the molecular mechanisms of heterosis. Indeed, high temperature-dependent weakness has been reported in interspecific rice hybrids and implicated two loci (*Hwi1*and *Hwi2*) as major determinants for this phenotype [8], suggesting existence of the phenomenon under a given condition even in crops with prevalent heterosis.

Nitrogen is a fundamental element for protein synthesis during plant growth and development [9–11] and is frequently limited under natural settings. Although nitrogen fertilizer has been widely used to compensate for nitrogen deficiencies in soil, its excessive application leads to increased cost and environmental pollution [12–14]. Thus, low-nitrogen tolerance, or higher nitrogen use efficiency, is a highly desirable trait for crop improvement [15, 16].

After NO_3_^-^ is biochemically reduced to NH_4_^+^, NH_4_^+^ is incorporated into organic molecules via glutamine synthetase (*GS*) and either glutamate synthase (*GOGAT*) or glutamate dehydrogenase (*GDH*) [17]. Glutamine synthetase (*GS; EC6*.*3*.*1*.*2*) primarily exists as two isozymes with different subcellular localizations: *GS1* in the cytosol and *GS2* in chloroplasts/plastids [18]. In rice, three genes *OsGS1;1*, *OsGS1;2* and *OsGS1;3* encode for *GS1* [19], of which *OsGS1;1* and *OsGS1;2* are especially abundant in the aerial tissues and roots, whereas *OsGS1;3* was specifically expressed in spikelet tissue [20].

Since the final product (glutamine) of the nitrogen metabolism pathway (NMp) is the initial substrate in chlorophyll (Chl) biosynthesis, it is critical to analyze the relevant genes in the porphyrin and Chl metabolic pathways (PCMp). Chl molecules exist ubiquitously in photosynthetic organisms and perform essential roles in harvesting light energy in the antenna systems and by driving electron transfer in the reaction centers [21]. Chl metabolism has been extensively studied with various plants using biochemical [22] and genetic [23–25] methods. The whole pathway from glutamate to Chl b is comprised of 16 steps in higher plants and can be subdivided into three parts: (*i*) formation of 5-aminolevulinic acid (ALA) from glutamate, (*ii*) formation of protoporphyrin IX from ALA, and (*iii*) formation of Chl from protoporphyrin IX. The genes involved in Chl biosynthesis in higher plants have been identified [26]. However, little is known about the differential expression of critical genes involved in these pathways between hybrids versus parental lines under normal or stressful conditions.

Here, we report that interspecific F1 hybrids between *Oryza sativa* ssp. *japonica* and a tetraploid wild rice species, *O*. *alta* exhibit hybrid weakness in several phenotypic and physiological traits compared with parental lines under normal or N-limiting conditions. We analyzed total expression levels and expression partitioning of parental alleles for critical genes involved in the NMp, PSp (photosynthesis pathway) and PCMp pathways. We reveal a strong correlation between altered expression level and/or biased parental allele expression partitioning of the analyzed genes and condition-specific hybrid weakness traits.

## Results

### Phenotypes of the F1 triploid hybrid plants of *O*. *sativa* and *O*. *alta* under normal and nitrogen-limiting conditions

**Fig 1:**
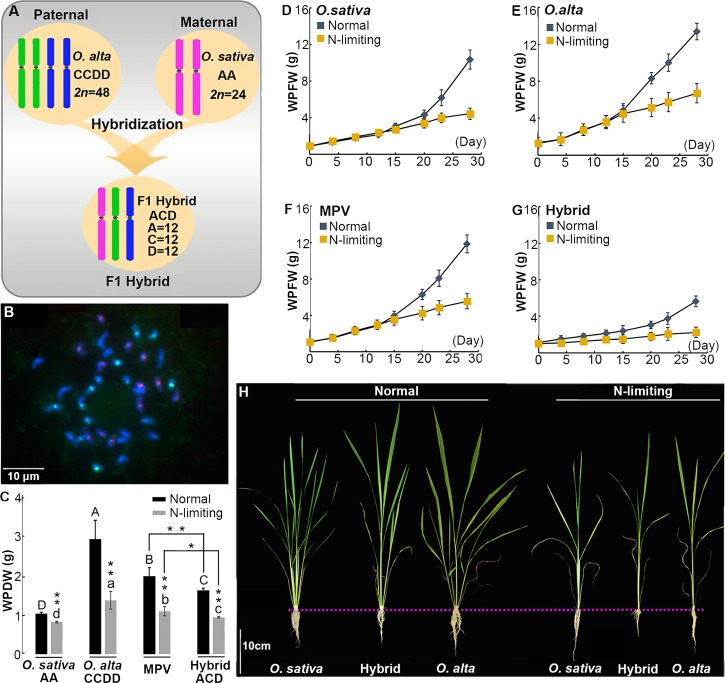
Cytology and typical phenotypes of the F1 triploid hybrid plants between *Oryza sativa* ssp. *japonica* (*2n* = 2x = 24, genome AA) and *O*. *alta* (*2n* = 4x = 48, genome, CCDD) under both normal and N-limiting conditions. (**A**) Diagrams showing genesis of the F1 triploid rice hybrids; (**B**) Typical chromosomal constitution of the triploid hybrids based on multicolor GISH analysis (Bar = 10 μm). The pink-colored chromosomes belong to the A genome, green-colored chromosomes are the C genome and blue-colored chromosomes are the D genome; (**C**) Whole plant dry weight of parental plants (MPVs) and F1 hybrids under normal and N-limiting conditions, WPDW stands for whole plant dry weight. Different letters among genotypes of the same treatment denote statistical differences (based on the least significant difference or LSD test, *P* < 0.05). The capital and small letters refer to corresponding indices of normal and N-limiting conditions, respectively, Error bars indicate s.d., n (replicates) = 3 to 4; (**D-G**) The growth rates and whole plant fresh weight of parental plants (MPVs) and F1 hybrids under normal and N-limiting conditions after the treatment for 4 weeks, the blue-dotted lines represent normal conditions and the yellow-dotted lines N-limiting conditions, WPFW stands for whole plant fresh weight, error bars indicate s.d., n (replicates) = 3 to 4; (**H**) Whole plant phenotypic differences between normal and N-limiting conditions at the final seedling stage (Bar = 10 cm).

A set of triploid hybrid plants (genome ACD, *2n* = 3x = 36) with *Oryza sativa* ssp. *japonica*, cv. Nipponbare (genome AA, *2n* = 2x = 24) as the maternal parent and a wild tetraploid rice species, *O*. *alta* (genome CCDD, *2n* = 4x = 48) as paternal parent [27], were used in this study (**Fig 1A**). The bona fide hybrid nature of all 30 plants used in this study was validated by genomic *in situ* hybridization (GISH) and chromosome counting (as exemplified in **Fig 1B**). All 30 hybrid plants were found to be karyotypically stable as no changes in either chromosome number or gross structure were detected at the GISH level, consistent with our previous results [27]. The hybrid plants showed overall intermediate status at the final seedling-stage under normal growing conditions compared to the aerial structures of the parental species (**Fig 1H**, left panel), implicating the overall lack of hybrid heterosis. On the other hand, the heading date of the F1 hybrids and the two parents are quite similar in our greenhouse environment, and the two parents, Nipponbare (*O*. *sativa*) and *O*. *alta*, are both fully fertile. In contrast, the F1 hybrids are completely sterile as expected for triploid F1 hybrids between distinct species [28, 29].

Here we sought to investigate whether performance of these hybrid plants would be different under stressful growing conditions compared to normal conditions. We chose N-limiting conditions because it is a moderate abiotic stress and is often experienced by crops grown in less fertile fields or with inefficient or insufficient nitrogen fertilizer input. We found that under both normal and N-limiting conditions, the F1 hybrid plants showed clear phenotypic weakness in several traits compared to the expected parental average (mid-parent value, MPV) at the final seedling-stage. The assayed phenotypic traits included: whole plant dry weight under the treatment for 4 weeks (**Fig 1C)**, whole plant fresh weight under the N-limiting treatment for 4 weeks (**Fig 1D–1G)** at the final seedling stage.

Quantification of these traits also showed inferior performance by the F1 hybrids relative to the MPVs (e.g., **Fig 1C, 1F vs. 1G**). We refer to this generally weakened performance of the F1 hybrids as hybrid weakness (in contrast to hybrid vigor or heterosis). Interestingly, when comparing the degrees of weakness in the F1 hybrids between normal and N-limiting conditions for these traits, we found that the hybrid weakness was clearly attenuated under N-limiting conditions. For example, although whole plant dry weight (WPDW) of the hybrid plants was significantly less than the corresponding MPVs under both normal and N-limiting conditions, the extent of decrease in the trait values was significantly smaller in the latter condition (*P* = 0.02) than in the former (*P* = 0.009) (**Fig 1C**). In addition, relative to normal conditions, F1 hybrids also showed attenuated hybrid weakness compared to corresponding MPVs in both whole plant fresh weight (WPFW) and growth rate under N-limiting conditions at a given time-point or growth stage (**Fig 1D–1G**). Specifically, under both conditions, F1 hybrids showed inferior performance compared with both parents (*O*. *sativa* and *O*. *alta*) and predicted MPVs in WPFW and growth rate across all the time-points analyzed; nevertheless, the differences between the two conditions were the smallest in the hybrids for the later (after 15 days) time points (**Fig 1D–1G**).

**Fig 2:**
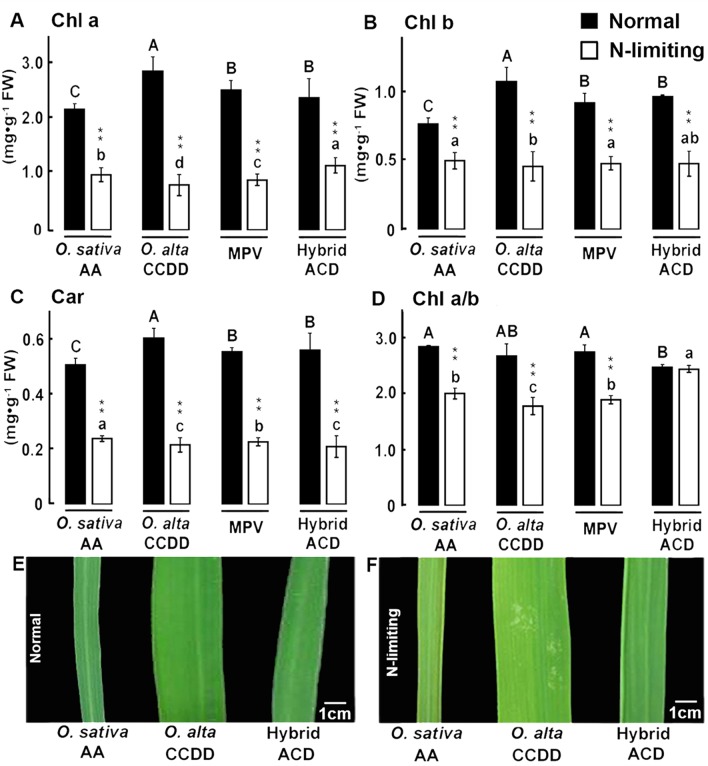
Photosynthetic pigment content from hydrolyzed leaves in parental plants (MPVs), and F1 hybrids under normal and N-limiting conditions. Pigment contents including: **(A)** Chl a content, **(B)** Chl b content, **(C)** Car content and **(D)** Chl a/b. Bar charts showing mean values of all replicates in each genotype. Black and while bars represent normal and N-limiting conditions, respectively. Different letters among genotypes of the same treatment denote statistical differences (by least significant difference or LSD test, *P* < 0.05). The capital and small letters refer to corresponding indices of normal and N-limiting conditions, respectively. Statistical differences in photosynthetic pigment contents for a given genotype between normal and N-limiting conditions are denoted by double asterisks (*P* < 0.01), error bars indicate s.d., n (replicates) = 9 to 12. (**E-F**) leaf color difference between parents and F1 hybrids under normal and N-limiting conditions.

Next, we investigated the physiological basis underlying leaf color differences between the hybrid plants and their parents under normal and N-limiting conditions. We measured Chl content in leaves, and found that under normal conditions, the content of Chl a, Chl b and Car (carotenoids) showed no difference between the hybrid plants and the corresponding MPVs (**Fig 2A–2C**), and only the ratio of Chl a/b content, a parameter for photosynthetic efficiency [30, 31], was significantly lower in hybrids than the corresponding MPVs (**Fig 2D**). In contrast, under N-limiting conditions, Chl a content and ratios of Chl a/b in hybrid plants were significantly higher than the corresponding MPVs, suggesting photosynthetic efficiency of the hybrid plants was significantly better than the mean of both parents (MPVs). Thus, F1 hybrids exhibited heterosis for these two traits under N-limiting conditions (**Fig 2A and 2D**), which is in accordance with the leaf color phenotype (**Fig 2E and 2F**).

### Attenuation of hybrid weakness under N-limiting conditions is accompanied by up-regulated expression of critical genes in photosynthetic and nitrogen metabolism pathways

To gain insights into the molecular basis underlying the attenuated hybrid weakness in biomass and Chl content, genes from three relevant pathways were analyzed by real-time qRT-PCR. The three related pathways were NMp, PSp and PCMp, and six, four and 11 genes were chosen from these three pathways, respectively. These 21 genes were also chosen because they were found in both parental lines (**S1 Table)** that allowed total expression levels to be assessed in the F1 hybrids and their parents (MPVs). We found that majority of the 21 analyzed genes showed down-regulation in the hybrids relative to MPVs under normal conditions, whereas the opposite trend, i.e., more up-regulated genes in hybrids vs. MPVs was observed under N-limiting conditions. Specifically, relative to the corresponding MPVs, four genes showed additive expression in the F1 hybrids, four genes showed up-regulation, and 13 genes showed down-regulation (**Table 1**). In contrast, under the N-limiting conditions, two genes showed additive expression, two genes showed down-regulation, and 17 genes showed up-regulation in the hybrids vs. MPVs (**Table 1**).

**Table 1:** Number and percentage (%) of genes showing various expression patterns under normal and N-limiting conditions.

Pathway	Normal			N-limiting		
	Additive	Non-additive		Additive	Non-additive	
		Up	Down		Up	Down
**NMp**	2	1	3	2	3	1
**PSp**	0	0	4	0	4	0
**PCMp**	2	3	6	0	10	1
**Total**	4	4	13	2	17	2
**(%)**	19.0	19.0	62.0	9.5	81.0	9.5

**Fig 3:**
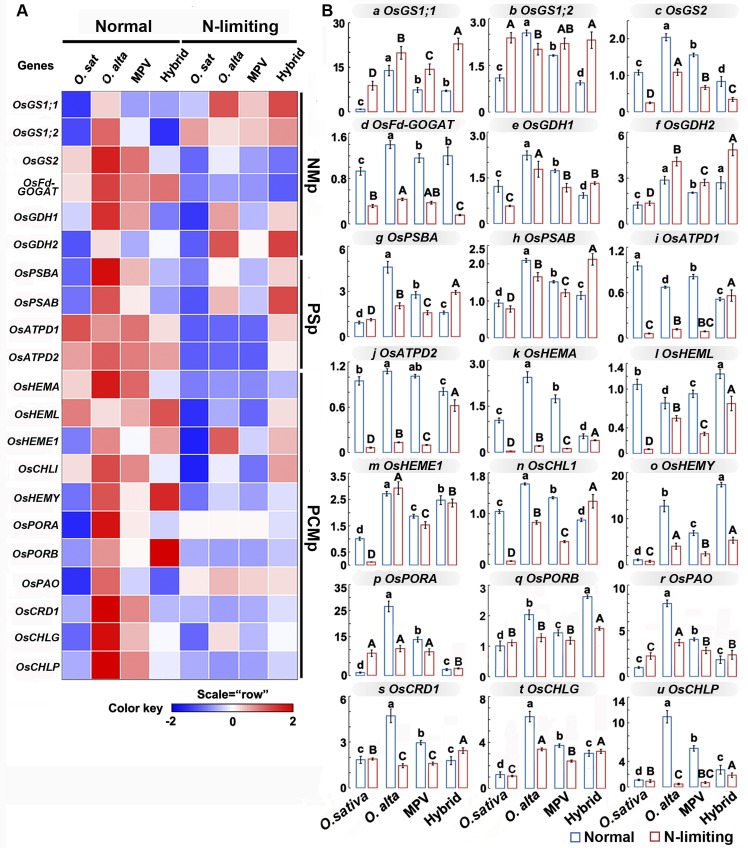
Gene expression profiling of 21 analyzed genes in F1 hybrids vs. *O*. *sativa* and *O*. *alta* parents under normal and N-limiting conditions. (**A**) Heat map showing expression level of the four replicated samples for all 21 genes assayed that are involved in the three pathways under normal vs. N-limiting conditions. Each row represents a single gene and the colors from red to blue denote expression levels from high to low. (**B**) Bar charts depicting the pairwise comparisons of gene expression level changes with error bars and statistical designations among four plant groups under normal vs. N-limiting conditions. Each bar chart represents a single gene, the bars in blue correspond to normal conditions and the bars in red correspond to N-limiting conditions. Different letters in the same treatment represent significant differences among samples based on the least significant difference (LSD) test (*P* < 0.05), capital and small letters refer to statistical differences between either normal or N-limiting conditions, respectively.

Next, we analyzed expression of the 21 genes in more detail. By comparing the F1 hybrids with the corresponding MPVs under normal vs. N-limiting conditions, these 21 genes could be grouped into three categories including six expression pattern groups (**Table 2**). Category I was termed "conserved genes", referring to genes that showed the same trend in expression pattern changes in F1 hybrids vs. MPVs under both normal and N-limiting conditions, which include two groups: (a) "both up-regulated" referring to genes that showed up-regulated expression in F1 hybrids relative to MPVs under both normal and N-limiting conditions, and (b) "both down-regulated", referring to genes that showed down-regulated expression in F1 hybrids relative to MPVs under both normal and N-limiting conditions. Category II was termed "positive responding genes" meaning that they showed higher expression levels in N-limiting vs. normal conditions, which include three groups: (c) "additive expression/up-regulation", referring to genes that showed additive expression in F1 hybrids relative to MPVs under normal conditions but up-regulation under N-limiting conditions, (d) "down-regulation/additive expression", referring to genes that showed down-regulation in F1 hybrids relative to MPVs under normal conditions but additive expression under N-limiting conditions, and (e) "down-regulation/up-regulation" referring to genes that showed down-regulated expression in F1 hybrids under control conditions but up-regulated expression under N-limiting conditions. Category III was termed "negative-response genes" meaning that they showed lower expression levels in N-limiting vs. normal conditions, which include one group (f) "additive expression/down-regulation", referring to genes that showed additive expression in F1 hybrids relative to MPVs under control conditions but down-regulated expression under N-limiting conditions. Notably, of the 21 genes studied, 13 (61.9%) belonged to Category II, most of which (nine out of 13) fell into expression pattern group e, which referred to genes that showed down-regulated expression in F1 hybrid under control conditions but up-regulated expression under N-limiting conditions (**Table 2 and Fig 3**). Therefore, the differential expression of these important genes involved in two of the three relevant pathways (PSp and PCMp) might be an important determinant at the total gene expression level that underlies the attenuation of the hybrid weakness phenotypes under N-limiting vs. normal conditions.

**Table 2:** Number and percentage (%) of genes in the three pathways showing changes in expression pattern in N-limiting versus normal conditions.

Category	I		II			III
Gene group	a	b	c	d	e	f
**NMp**	1	1	1	2	0	1
**PSp**	0	0	0	0	4	0
**PCMp**	4	0	1	0	5	1
**Total no.**	5	1	2	2	9	2
**(%)**	23.8	4.8	9.5	9.5	42.9	9.5

**Note:** (1) I, II and III, refer to the three categories, i.e., conserved, up-regulation during N-limitation and down-regulation during N-limitation. (2) The a, b, c, d, e and f refer to gene expression pattern groups of both up-regulated (a), both down-regulated (b), additive expression/up-regulation (c), down-regulation/additive expression (d), down-regulation/up-regulation (e), and additive expression/down-regulation (f), in the normal vs. N-limiting conditions, respectively. For example, group "e" (belonging to category II) means that some genes in F1 hybrids showed down-regulation under normal conditions but up-regulation under N-limiting conditions relative to the corresponding MPVs.

### Attenuation of hybrid weakness under N-limiting conditions is also accompanied by increased maternal allele partitioning of critical genes

**Fig 4:**
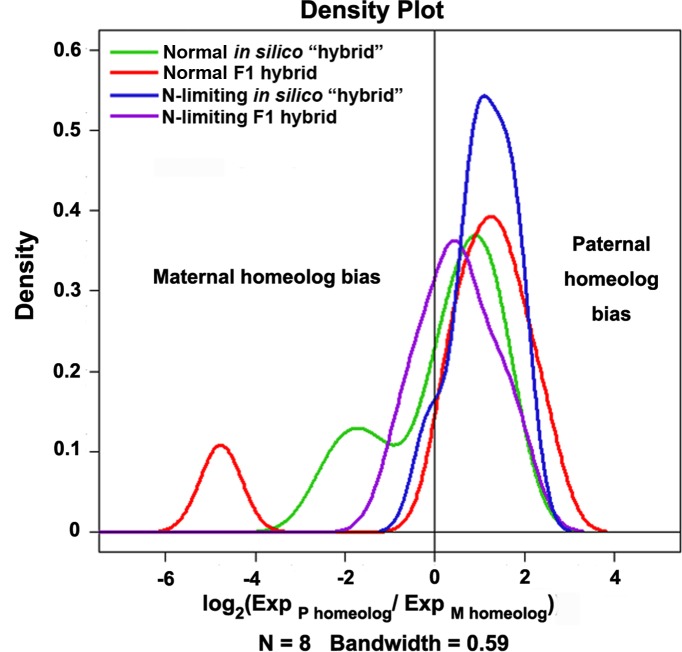
Density distribution plot showing parental allele expression partitioning for eight analyzed genes by cDNA-pyrosequencing in the F1 hybrids vs. *in silico* "hybrids" (parental cDNA mix) under normal and N-limiting conditions. Green and blue lines represent the density of in silico “hybrid” under normal and N-limiting conditions, respectively. Red and pink lines represent the density of F1 hybrid under normal and N-limiting conditions, respectively. X-axis is log_2_ of expression ratio between paternal homeolog to maternal homeolog for each tested gene, “0” on x-axis stands for parental homeologs expressed equally. Gene number N = 8, the bandwidth stands for the standard deviation of the kernel and a measure of how closely you want the density to match the distribution, which means when the bandwidth is 0.59, the density curve will be smooth.

It has been widely shown in diverse plant hybrids and allopolyploids (hybrid polyploids) that while alteration in total expression levels in hybrids contributes to heterosis, variable partitioning of parental alleles also plays a role [32–34]. In light of this generic finding and given that most of the 21 genes we analyzed showed altered total expression levels in the hybrids vs. the corresponding MPVs or between the two conditions (normal vs. N-limiting), described above, we asked whether the parental alleles contributed proportionally or in a biased manner to the cumulated changes in total expression level of these genes. For this purpose, we employed cDNA-pyrosequencing [35] on eight of the 21 genes studied using primers specially designed for pyrosequencing (**S2 Table**). Using this analysis, we quantified parental allelic expression contributions to transcript pools of the F1 hybrids under either normal or N-limiting conditions. It should be noted that, in both *in silico* "hybrids" and F1 hybrids, a two-fold increase in paternal allele expression was expected given the 1:2 subgenome contributions by the two parental species (**Fig 1**). Bearing this in mind, we conducted all possible pairwise comparisons between the both the normal N-limiting conditions for a given sample (*in silico* "hybrids" or F1 hybrids) and between samples for a given condition, and presented the data as a density plot (**Fig 4**). Importantly, we found that greater than expected maternal allele expression occurred in the *in silico* "hybrids" than the F1 hybrids under normal conditions (compare the green line and the red line in **Fig 4**), and in F1 hybrids under N-limiting conditions than in F1 hybrids under normal conditions (compare the pink line and the red line in **Fig 4**). Taken together with the phenotypic results showing that the parents (MPVs) performed better than the F1 hybrids (hybrid weakness) under normal conditions, and F1 hybrids performed better in N-limiting conditions than F1 hybrids in normal conditions (attenuated hybrid weakness), led to the conclusion that biased maternal allele partitioning is likely a contributing factor to the attenuation of hybrid weakness under N-limiting conditions.

### Physiological and morphological features of F1 hybrids show more resemblance to the *O*. *sativa* maternal parent under N-limiting vs. normal conditions

**Fig 5:**
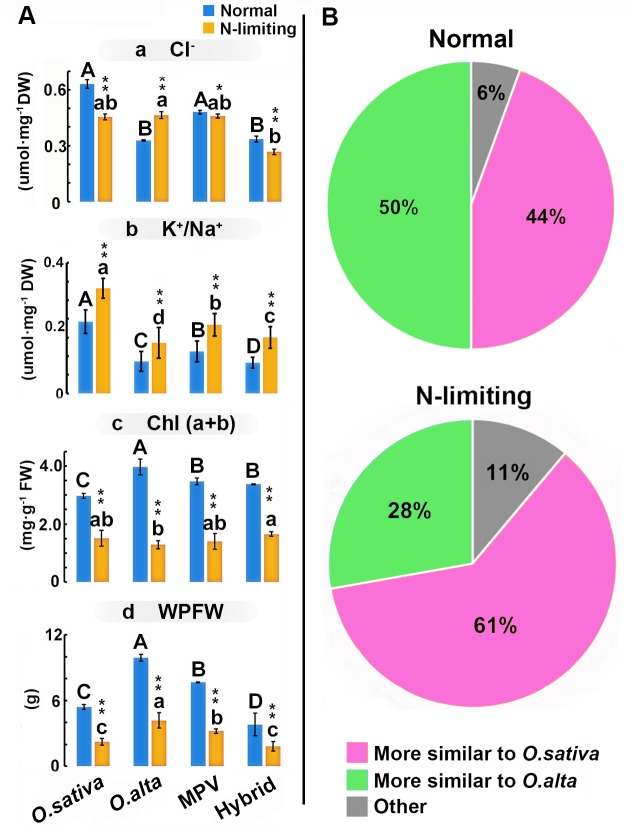
Overall resemblance of the F1 hybrids to *O*. *sativa* (maternal) or *O*. *alta* (paternal) parents, in 18 measured physiological or morphological traits under normal and N-limiting conditions. (**A**) Bar charts represent physiological or morphological traits, including (a) Cl^-^ concentration, (b) the ratio of the concentration K^+^ and Na^+^, (c) Chl (a+b) content, (d) whole plant fresh weight, the blue bars represent normal conditions and the orange bars represent N-limiting conditions. Different letters under same conditions represent significant difference among the samples. Capital and small letters denote for normal and N-limiting conditions, respectively. * and ** above the orange column refer to significant (*P* < 0.05) or extremely significant (*P* < 0.01) differences (based on LSD test) between the two conditions for a given physiological trait of each sample (i.e., comparisons between each pair of blue and orange columns). Error bars indicate s.d., n (replicates) = 3. (**B**) The similarities were depicted based on quantification of each of the 18 physiological or morphological traits as described by the methods of Yang et al. [36].

We chose 18 physiological and morphological traits to measure since they are related to N-metabolism and photosynthetic pigment contents and compared the differences in these 18 physiological and morphological traits between the F1 hybrids and the *O*. *sativa* and *O*. *alta* parents (reflected by MPVs) under normal and N-limiting conditions. We observed contrasted differences between the hybrids and the corresponding MPVs for many of these traits between the normal and N-limiting conditions (**Figs 2A–2C and 5A and S2 Fig**). For example, NO_3_^-^ content was significantly lower in hybrids than the MPVs; however, under N-limiting conditions, the difference was no longer significant (**S2A Fig**). Likewise, under normal conditions, there was no significant difference between hybrids and the MPVs in all three photosynthetic pigments (Chl a, Chl b and Car); under N-limiting conditions, however, hybrids contained significantly more Chl a and less Car (**Fig 2A–2C**). Also, the Chl a/b ratio was significantly lower in hybrids than MPVs under normal conditions, but was significantly higher in hybrids than MPVs under N-limiting conditions (**Fig 2D**). The last observation suggests that hybrid weakness in photosynthetic efficiency under normal conditions transitioned to hybrid vigor in the N-limiting conditions.

We next asked the question whether the contrasted differences in many of these physiological and morphological traits between the normal and N-limiting conditions (**Figs 2A–2C and 5A and S2 Fig**) may cause more or less of a phenotypic resemblance of the hybrids to either of the parents, *O*. *sativa* (maternal) or *O*. *alta* (paternal). Taking all 18 physiological and morphological traits together, we found that the hybrids showed similar resemblance to either parent (44% vs. 50%) under normal conditions, but more resemblance to the maternal parent (*O*. *sativa*) than the paternal parent (*O*. *alta*) (61% vs. 28%) under N-limiting conditions (**Fig 5**). This is consistent with the DNA-pyrosequencing data indicating an increase in partitioned maternal expression in the hybrids under N-limiting conditions vs. normal conditions (**Fig 4**).

## Discussion

Hybrid vigor or heterosis is one of the most fascinating manifestations of hybridization. Although heterosis has been intensively studied due to its enormous benefit to crop production, the mechanistic underpinnings of the phenomenon remain elusive [4]. Comparatively much less attention has been paid to hybrid weakness than the opposite phenomenon of heterosis. We asserted that a better understanding of hybrid weakness may shed novel insights into the mechanisms of heterosis.

In the course of our efforts to make wide hybrids between distantly related species in the genus *Oryza*, we noted that F1 hybrids between *O*. *sativa*, ssp. *japonica* (genome AA) and a tetraploid wild rice species, *O*. *alta* (genome, CCDD) exhibited apparent hybrid weakness in a suite of morphological and physiological traits. Furthermore, we found that the extent of hybrid weakness was significantly attenuated if the hybrids were grown under nitrogen-limiting conditions. To explore whether the phenotypic alternation might be related to alterations in gene expression, we selected 21 key genes involved in three important pathways pertinent to the assayed morphologic and physiological traits, and quantified their expression level by qRT-PCR for the hybrids under both normal and N-liming conditions. In addition, we quantified parental allele partitioning of a subset of these 21 genes (that met the stringent requirement for this assay) by locus-specific cDNA-pyrosequencing. Overall, our results suggest that both altered gene expression level and differential use of parental alleles of critical genes may plausibly explain hybrid weakness and its condition-dependent attenuation observed in the F1 rice hybrids.

A prevailing model to explain heterosis proposes that deleterious parental alleles are compensated for in hybrid progeny by a cumulative enhancement of gene expression [37, 38]. This model apparently cannot explain the existence of hybrid weakness. A previous study in maize documented cases of positive associations between robust gene expression and superior phenotypes in hybrids [39], which is inclusive with the occurrence of hybrid weakness because robust gene expression can be genotype and/or condition-dependent. Recently, quantitative novel gene expression, which is deviated from mid-parent values (non-additive expression), has been suggested as a major determinant of heterosis [40, 41]. In addition, epigenetic modification of critical genes in the circadian pathway resulting in their altered expression was found to be causal to heterosis in an Arabidopsis allotetraploid [42]. The above studies have emphasized the link between altered gene expression in the hybrids and heterosis. Whether changes in gene expression are also related to hybrid weakness remains unknown although it is clear that the phenotype of hybrid weakness is also genetically determined [8].

The 21 genes we chose to study are from three pathways, nitrogen metabolism, photosynthesis, prophyrin and chlorophyll metabolism, which are crucial for rice growth and development and relevant to the morphological and physiological phenotypes we assessed for hybrid weakness. Overall, we found that N-limiting conditions resulted in rapid down-regulation of genes in the photosynthetic and Porphyrin and Chl Metabolism (PSp and PCMp) pathways, which is in accordance with a previous study showing that genes involved in photosynthesis and energy metabolism are rapidly down-regulated during N-limitation that underlies the poor plant growth [15]. We thus speculate that the weakness in the rice F1 hybrids under normal conditions may result from the down regulation of corresponding genes in Chl and photosynthetic pathways (**Fig 3**). Thus, our observation that 42% of the genes studied that showed down-regulated expression in the F1 hybrids under normal conditions reverted to up-regulated expression under the N-limiting conditions is striking. Moreover, our results showed that 13 of the 21 genes in the hybrids under N-limiting conditions were up-regulated not only relative to MPV, but also transgressively (two of six in NMp, four of four in PSp and seven of 11 in PCMp). Importantly, we found that this reverted gene expression from down- to up-regulation is concomitant with the reduced weakness of the F1 hybrids (**Table 2 and Fig 3**). Taken together, our results strongly suggest a link between altered gene expressions of critical genes to hybrid weakness, as has been documented for heterosis.

In a hybrid genome, total gene expression level is not the only aspect of gene expression, as differential use of parental alleles also plays a role. Our quantitative results on eight of the 21 genes by locus-specific cDNA-pyrosequencing indicated that the degree of maternal allele contribution to the transcript pool of the hybrids was significantly increased under N-limiting conditions than normal conditions, suggesting unequal parental allele contribution in response to N-limitation conditions in the hybrids, which again is concomitant with the attenuation of hybrid weakness. Taken together, our results suggest that both changes in total expression level of critical genes as well as differential partitioning of parental alleles contribute to the extent of hybrid weakness in the rice hybrids, and which is variable depending on environmental conditions. Further experiments are needed to gain a more comprehensive understanding of hybrid weakness vs. vigor under different conditions and to shed light on its molecular underpinnings.

## Materials and methods

### Plant materials and growth conditions

The interspecific rice hybrids were produced by crossing the tetraploid wild species of *Oryza* (*O*. *alta*, genome CCDD, IRGC Acc. No. 100161) as the paternal parent with the standard laboratory rice cultivar Nipponbare (*O*. *sativa* ssp. *japonica*, genome AA) as maternal parent and embryo rescue was subsequently employed [43]. The original *O*. *alta* seeds were kindly provided by Dr. Song Ge at the Institute of Botany, Chinese Academy of Sciences. The accessions were extensively selfed more than eight times to ensure homozygousity before the crosses were made. The seedlings grown in a growth chamber that was maintained at 27.0±1.5°C during the day and 22.0±1.5°C during the night. The nutrient solution used in this work is described by the International Rice Research Institute [44], and contained 1.44 mM NH_4_NO_3_, 0.32 mM NaH_2_PO_4_, 0.6 mM K_2_SO_4_, 1.0 mM CaCl_2_, 1.6 mM MgSO_4_, 0.072 mM Fe-EDTA, 0.2 mM Na_2_SiO_3_, 9.1mM MnCl_2_, 0.154 mM ZnSO_4_, 0.156 mM CuSO_4_, 18.5 mM H_3_BO_3_ and 0.526 mM H_2_MoO_4_ at pH = 5.2. The nutrient solution was replaced daily.

### N-limiting treatment

Three materials of genome AA, ACD, CCDD were placed in floating trays and grown in nitrogen-based nutrition liquid media until the five-leaf stage. Eighteen to thirty-five individuals were selected to perform the N-limiting stress experiments. For each set of materials, two groups were designated as either control or the N- stress treatment. The concentration of NH_4_NO_3_ in N-limiting nutrient solution was lowered to 0.048 mM, which was 1/30th of the nitrogen supplied to the control. Three biological replicates with three to four seedlings from each genotype per replicate were used under the two treatment solutions. All the seedlings of each genotype under the two treatment solutions were harvested after four weeks of treatment.

### Measurement of photosynthetic pigments

After 4 weeks treated with N-limiting nutrient solution, there were nine to twelve seedlings for each genotype. Chl a, Chl b and carotenoids were measured in the same part of the leaves at the same developmental stage individually, which followed the method of Zhu [45], and expressed in mg·g^-1^ FW. The data analysis was performed using a Student’s t-test.

### Biomass determination and anion content analysis

After four weeks of the N-limiting treatment, plant materials were harvested from each genotype under both control and N-limiting conditions for biomass determination and biochemical analysis. Shoots/seedlings of all genotypes were collected and assayed. For each condition, nine to twelve individuals in each genotype were used in the biomass determination. For anion content measurements, three to four seedlings were pooled for each genotype in order to obtain three biological replicates and triturated in each replicate. The drying procedure was performed using the following protocol of 15min at 95°C followed by 24h at 55°C. Dry samples of the plant material were suspended in 10 mL of deionized water maintained for 48h at 100°C, and the extracts were used to determine the contents of free inorganic anions. The contents of NO_3_^–^, Cl^–^, H_2_PO_4_^–^, and SO_4_^2–^ were determined by ion chromatography with the DX-300 ion chromatographic system (Thermal Scientific) using an AS4A-SC ion-exchange column and a CD M-II electrical conductivity detector (Dionex). The mobile phase consisted of Na_2_CO_3_/NaHCO_3_ = 1.7/1.8 mM.

### Physiological and morphological traits analyses of F1 hybrids

For the analysis of overall resemblance by the F1 hybrids to either parent, the similarities were depicted based on quantification of each of the 18 physiological or morphological traits by the method of Yang et al. [46]. Differences between *O*. *sativa* and F1 hybrids were calculated by D2 = (*O*. *sativa*—Hybrid)/Hybrid ×100%, and differences between *O*. *alta* and F1 hybrids were calculated by D4 = (*O*. *alta*—Hybrid)/ Hybrid × 100%. Thus, for a given physiological or morphological trait, the difference (D = |D2|−|D4|) between D2 and D4 indicates whether the hybrid was more similar to *O*. *sativa* or to *O*. *alta*. Thus, |D2|>|D4| (D > 5%) indicates hybrid is more similar to *O*. *alta* than *O*. *sativa*; conversely, |D2|<|D4| (D < 5%) indicates hybrid is more similar to *O*. *sativa* than *O*. *alta*. For those traits in which D is in the range of −5% to +5%, it denotes all other situations, such as the hybrid is similar to neither *O*. *sativa* nor *O*. *alta* or similar to both parents.

### RNA extraction

For each genotype (*O*. *sativa*, *O*. *alta* and F1 hybrid) under both normal and N-limiting conditions, a small piece of the second to last leaf was harvested independently from three randomly chosen individuals under the same developmental stage after four weeks of the N-limiting treatment (thus eighteen samples in total). All leaf materials were frozen with liquid nitrogen and then stored in -70°C freezer. Total RNA was isolated using the Trizol Reagent (Invitrogen) method from each biological sample, following the manufacturer’s protocol.

### Selection of genes for quantitative PCR and locus-specific pyrosequencing analysis

We first selected 26, 77 and 35 genes located in three critical growth relative pathways (Nitrogen Metabolism pathway, http://www.kegg.jp/kegg-bin/highlight_pathway?scale=1.0&map=map00910&keyword=Nitrogen Metabolism pathway; Photosynthesis pathway, http://www.genome.jp/kegg-bin/show_pathway?map00195 and Porphyrin and Chl Metabolism, http://www.kegg.jp/kegg-bin/highlight_pathway?scale=1.0&map=map00860&keyword=Porphyrin and Chl Metabolism pathway) as candidate genes for genotyping, by aligning gene sequences of Nipponbare with other distantly-related species using NCBI blastn. The conserved gene sequences of 36 genes were confirmed. Next, by designing primers in conserved sequences of these 36 confirmed genes, there were only 21 genes could be cloned from the *O*. *alta* CCDD tetraploid parent. The DNA sequences of all the primers we used for cloning the corresponding orthologous genes are listed in **S3 Table**. Among these 21 genes, only 15 genes contained solid SNPs between parents for distinguishing the two parental alleles/homeologs in pyrosequencing. Pyrosequencing primers were designed for all 15 genes, and only eight genes were successfully used in pyrosequencing (**S2 Table**).

### Quantitative PCR and locus-specific cDNA pyrosequencing

Real-time quantitative (q)PCR and pyrosequencing were performed to analyze the total gene expression level and allelic gene expression. Five micrograms of total RNA was isolated in triplicate samples of each genotype under both conditions and reverse transcribed to cDNA using the Super Script first strand synthesis kit (Invitrogen) according to the manufacturer’s instructions. Real-time quantitative (q)PCR amplification was performed with SYBR Green Real-Time PCR Master Mix reagent (Toyobo) on a Step One Plus Real-Time PCR apparatus (Applied Biosystems), and the amplification conditions were as follows: 95°C for 1 min, followed by 40 cycles of 2-step cycle of 95°C for 5 s and 60°C for 1 min. Primers for all analyzed genes were designed by the pyrosequencing system (PyroMark ID Q96; Biotage). Amplification of the rice UBQ5 (GenBank Accession AK061988) mRNA was used as an internal quantitative control [46–48]. The relative expression of the target genes was calculated using the ΔΔCt method [49]. Primers are listed in **S4 Table**. In cDNA Pyrosequencing analysis, RNA samples for *in silico* “hybrids” were made with mixture of parental RNA by Nipponbare:*O*. *alta* = 1:2. The detailed pyrosequencing protocol was essentially as reported by Mochida et al. [35] with modifications detailed by Zhang et al. [50]. Primer sequences of the eight genes used in pyrosequencing were also listed in **S4 Table**. Biotin labeled PCR products were immobilized on streptavidin-coated paramagnetic beads. Capture of biotinylated single-stranded PCR products, annealing of the sequencing primer, and solid-phase pyrosequencing were performed following the manufacturer’s recommendations.

### Statistical analysis

Statistical analysis was performed using the statistical program SPSS 13.0. Statistical significance was determined by Student’s t-test by *P* < 0.05 or least significant difference (LSD).

## Supporting information

S1 Fig An example of a pyrosequencing profile for the *OsGS2* gene. A diagnostic SNP (A vs. G) was identified between *O*. *sativa* and *O*. *alta* for this gene. For each of the samples (*in silico* "hybrids" and F1 hybrids) three biological replicates (marked as 1, 2 and 3) were included for each condition (control and N-limiting).
(TIF)

S2 Fig Quantification of 14 physiological and morphological traits in F1 hybrids and their parents, under normal and N-limiting conditions. Each bar represents a single physiological or morphological trait, including (**A-F**) anion or cation concentration, (**G, H**) the ratio of the photosynthetic pigment contents, (**I**) water content, blue bars represent normal condition and the orange bars represent N-limiting condition. Different letters under the same condition represent significant difference among the samples. Capital and small letters denote for normal and N-limiting conditions, respectively. * and ** above the orange column refer to significant (*P* < 0.05) or extremely significant (*P* < 0.01) differences (based on LSD test) between the two conditions for a given physiological trait of each sample (i.e., comparisons between each pair of blue and orange columns). Error bars indicate s.d., n (replicates) = 3. Chl shorts for chlorophyll; Car, carotenoid; Wc, water content.
(TIF)

S1 Table Detailed information for the 21 genes used in the study. (DOCX)

S2 Table Summary of parental contribution (%) to transcript pools of the *in silico* “hybrids” and F1 hybrids under normal and N-limiting conditions, based on gene-specific cDNA pyrosequencing. (DOCX)

S3 Table Primers used for amplifying genes in *O*. *alta*. (DOCX)

S4 Table Primers used in real-time qRT-PCR and pyrosequencing. Note: a, primers used in real-time qRT-PCR; b, primers used in pyrosequencing; bio, biotinylated primer.
(DOCX)
