# Supplementary figures and images for: Hybrid weakness in a rice interspecific hybrid is nitrogen-dependent, and accompanied by changes in gene expression at both total transcript level and parental allele partitioning
Source: PLoS One. 2017 Mar 1;12(3):e0172919. doi: 10.1371/journal.pone.0172919 (PMC5332110; doi:10.1371/journal.pone.0172919)

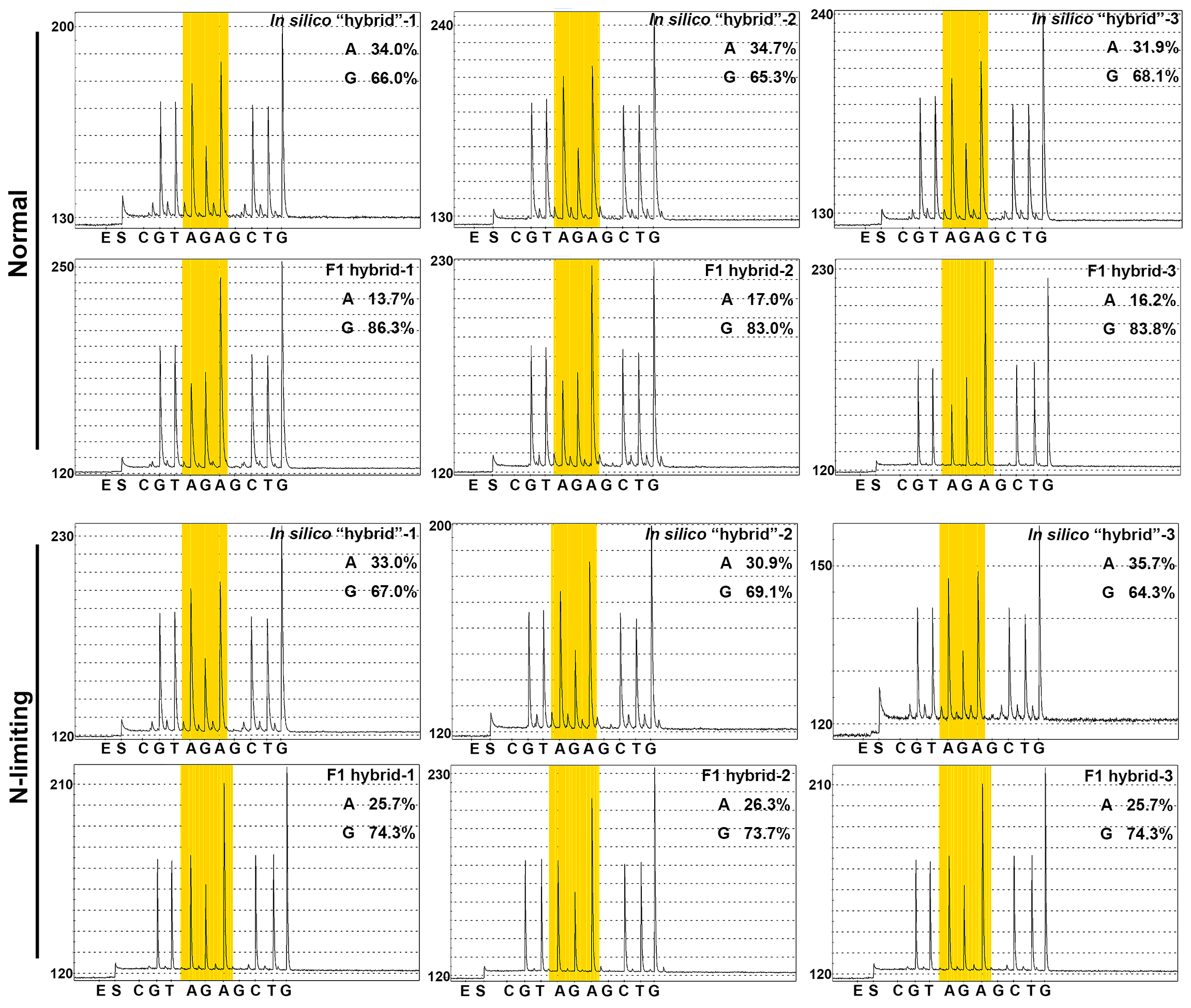

Supplement: S1 Fig — A diagnostic SNP (A vs. G) was identified between O. sativa and O. alta for this gene. For each of the samples (in silico "hybrids" and F1 hybrids) three biological replicates (marked as 1, 2 and 3) were included for each condition (control and N-limiting). (TIF) [file pone.0172919.s001.tif]

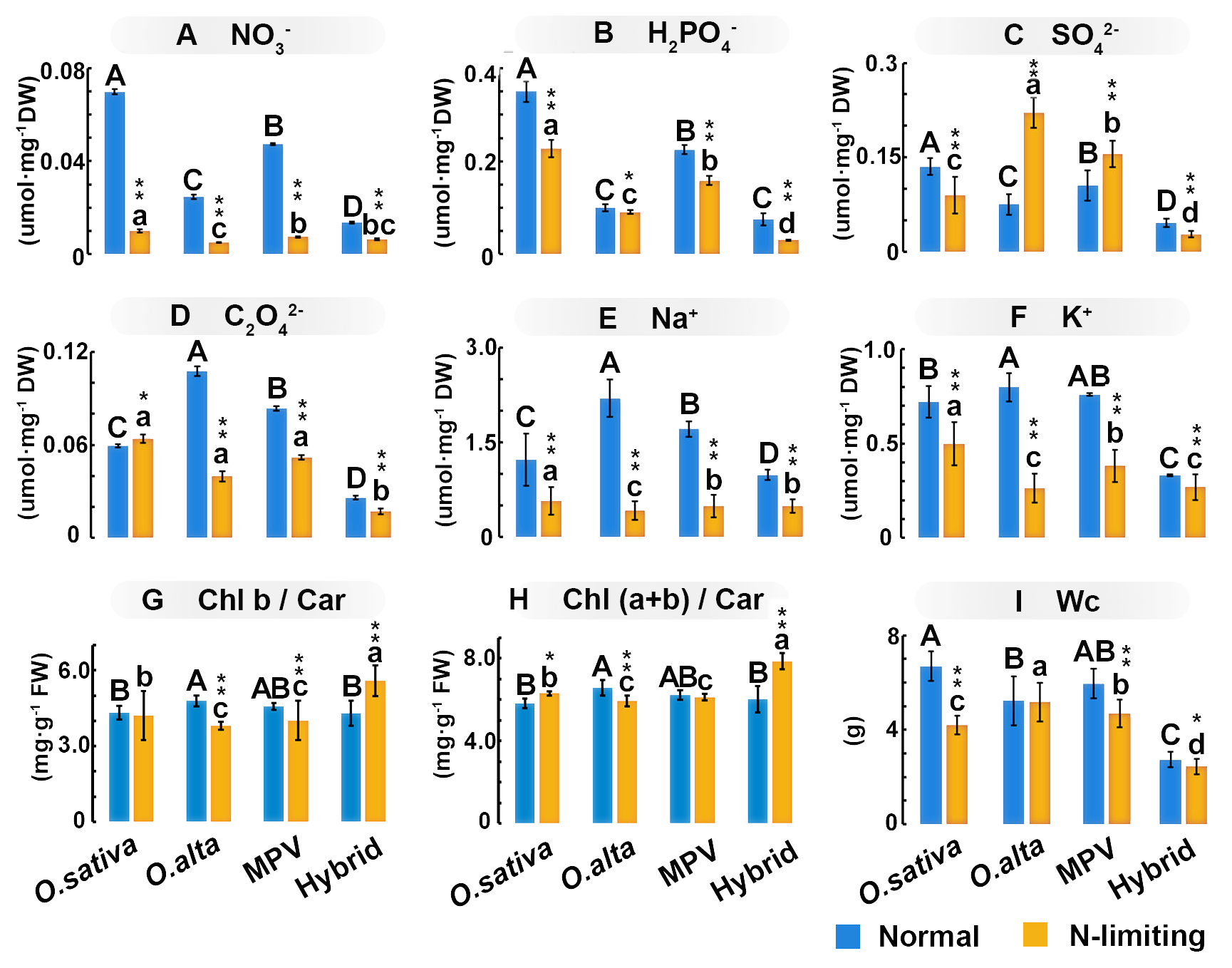

Supplement: S2 Fig — Each bar represents a single physiological or morphological trait, including (A-F) anion or cation concentration, (G, H) the ratio of the photosynthetic pigment contents, (I) water content, blue bars represent normal condition and the orange bars represent N-limiting condition. Different letters under the same condition represent significant difference among the samples. Capital and small letters denote for normal and N-limiting conditions, respectively. * and ** above the orange column refer to significant (P < 0.05) or extremely significant (P < 0.01) differences (based on LSD test) between the two conditions for a given physiological trait of each sample (i.e., comparisons between each pair of blue and orange columns). Error bars indicate s.d., n (replicates) = 3. Chl shorts for chlorophyll; Car, carotenoid; Wc, water content. (TIF) [file pone.0172919.s002.tif]
