# Supplementary material for: Hybrid weakness in a rice interspecific hybrid is nitrogen-dependent, and accompanied by changes in gene expression at both total transcript level and parental allele partitioning
Source: PLoS One. 2017 Mar 1;12(3):e0172919. doi: 10.1371/journal.pone.0172919 (PMC5332110; doi:10.1371/journal.pone.0172919)
Supplement: S1 Table — (DOCX) [file pone.0172919.s003.docx]

**S1 Table Detailed information for the 21 genes used in this study**

| **Gene Name** | **Locus ID** | **Function or putative function** |
| --- | --- | --- |
| *OsGS1;1* | LOC_Os02g50240 | Cytosolic glutamine synthetase 1;1 |
| *OsGS1;2* | LOC_Os03g12290 | Cytosolic glutamine synthetase 1;2 |
| *OsGS2* | LOC_Os04g56400 | Chloroplastic glutamine synthetase |
| *OsFd-GOGAT* | LOC_Os07g46460 | Ferredoxin dependent-glutamate synthase Fd-Gogat |
| *OsGDH1* | LOC_Os03g58040 | Glutamate dehydrogenase1 |
| *OsGDH2* | LOC_Os04g45970 | Glutamate dehydrogenase2 |
| *OsPSBA* | LOC_Os04g16770 | Photosystem II P680 reaction center D1 protein |
| *OsPSAB* | LOC_Os12g37040 | Photosystem I P700 chlorophyll a apoprotein A2 |
| *OsATPD1* | LOC_Os03g55874 | F-type H+-transporting ATPase subunit beta |
| *OsATPD2* | LOC_Os12g10570 | F-type H+-transporting ATPase subunit beta |
| *OsHEMA* | LOC_Os10g35840 | Glutamyl-tRNA reductase |
| *OsHEML* | LOC_Os08g41990 | Glutamate-1-semialdehyde 2,1-aminomutase |
| *OsHEME1* | LOC_Os01g43390 | Uroporphyrinogen decarboxylase |
| *OsCHLI* | LOC_Os03g36540 | Magnesium chelatase subunit I |
| *OsHEMY* | LOC_Os01g18320 | Oxygen-dependent protoporphyrinogen oxidase |
| *OsPORA* | LOC_Os04g58200 | Protochlorophyllide reductase |
| *OsPORB* | LOC_Os10g35370 | Protochlorophyllide reductase |
| *OsPAO* | LOC_Os03g05310 | Pheophorbide a oxygenase |
| *OsCRD1* | LOC_Os01g17170 | Magnesium-protoporphyrin IX monomethyl ester cyclase |
| *OsCHLG* | LOC_Os05g28200 | Chlorophyll synthase |
| *OsCHLP* | LOC_Os02g51080 | Geranylgeranyl reductase |
