# Supplementary material for: Hybrid weakness in a rice interspecific hybrid is nitrogen-dependent, and accompanied by changes in gene expression at both total transcript level and parental allele partitioning
Source: PLoS One. 2017 Mar 1;12(3):e0172919. doi: 10.1371/journal.pone.0172919 (PMC5332110; doi:10.1371/journal.pone.0172919)
Supplement: S2 Table — (DOCX) [file pone.0172919.s004.docx]

**S2 Table Summary of parental contribution (%) to transcript pools of the *in silico* “hybrids” and F1 hybrids under normal and N-limiting conditions, based on gene-specific cDNA pyrosequencing**

| **Gene name** | **Parental SNPs** | | **Control** | | **N-limiting** | |
| --- | --- | --- | --- | --- | --- | --- |
|  |  |  | ***In silico* “hybrids”** | **F1 hybrids** | ***In silico* “hybrids”** | **F1 hybrids** |
| *OsGS2* | *O. sativa* | A | 33.53% | 15.63% | 33.20% | 25.90% |
|  | *O. alta* | G | 66.47% | 84.37% | 66.80% | 74.10% |
| *OsGDH2* | *O. sativa* | C | 28.43% | 26.50% | 24.67% | 40.03% |
|  | *O. alta* | T | 71.57% | 73.50% | 75.33% | 59.97% |
| *OsPSAB* | *O. sativa* | A | 36.00% | 96.47% | 35.67% | 100.00% |
|  | *O. alta* | G | 64.00% | 3.53% | 64.33% | 0.00% |
| *OsHEMA* | *O. sativa* | T | 82.03% | 39.43% | 38.40% | 54.47% |
|  | *O. alta* | C | 17.97% | 60.57% | 61.60% | 45.53% |
| *OsHEME1* | *O. sativa* | T | 27.10% | 28.00% | 20.87% | 41.63% |
|  | *O. alta* | C | 72.90% | 72.00% | 79.13% | 58.37% |
| *OsCHLI* | *O. sativa* | C | 45.37% | 33.67% | 30.33% | 43.63% |
|  | *O. alta* | G | 54.63% | 66.33% | 69.67% | 56.37% |
| *OsPORB* | *O. sativa* | G | 48.47% | 21.23% | 23.60% | 22.50% |
|  | *O. alta* | A | 51.53% | 78.77% | 76.40% | 77.50% |
| *OsPAO* | *O. sativa* | T | 71.37% | 44.97% | 52.20% | 61.83% |
|  | *O. alta* | C | 28.63% | 55.03% | 47.80% | 38.17% |
