# Supplementary material for: Hybrid weakness in a rice interspecific hybrid is nitrogen-dependent, and accompanied by changes in gene expression at both total transcript level and parental allele partitioning
Source: PLoS One. 2017 Mar 1;12(3):e0172919. doi: 10.1371/journal.pone.0172919 (PMC5332110; doi:10.1371/journal.pone.0172919)
Supplement: S3 Table — (DOCX) [file pone.0172919.s005.docx]

**S3 Table Primers used for amplifying genes from *O. alta***

| **Gene name** | **Forward primer Sequence (5’-3’)** | **Reverse primer Sequence(5’-3’)** |
| --- | --- | --- |
| *OsGS1;1* | ggatcggtggatctggcatg | tgccgttctgctccgtctcc |
| *OsGS1;2* | gccgagtacatctgggttg | ggttcgccacgccccattt |
| *OsGS2* | gccgagtacatctgggttgg | gtttgccacaccccatgagaa |
| *OsFd-GOGAT* | gcttgtggtgttggatttgtcg | tgatcgcatccagttcaagtt |
| *OsGDH1* | gagaagagcttgctcatcc | aaggcgcccatgcggaggt |
| *OsGDH2* | ggcctcgactccaagctcg | gaatgttctgaacccactcaaa |
| *OsPSBA* | gtcgcttctgcaactggat | gagggaagttgtgagcatt |
| *OsPSAB* | ttccttagtagctcaacat | caagcagaaatatcacaag |
| *OsATPD1* | aagtgattgacacgggagc | ctttcgtgctagcttcatc |
| *OsATPD2* | cgagttagatacgaaatta | cagtttcataatgttcgttg |
| *OsHEMA* | gttgcagaggaactatggc | gcttccacaacctctttca |
| *OsHEML* | gattatgttggttcctggg | tgtggattccagcagtcat |
| *OsHEME1* | catggcggagtaccaggcg | ctgtccagtcaagaccaat |
| *OsCHLI* | gggcaggacgagatgaagc | tggcagccctgttagtcac |
| *OsHEMY* | gaggaggggcccaacagct | ttacagcagcaactggtgg |
| *OsPORA* | aagtggcacgtggtgatgg | gctgtccttgtacgccttg |
| *OsPORB* | ccgacggcttcgagatgag | ttccagctccagtacaccc |
| *OsPAO* | ccccgttccagctcctcaa | agcaaatccatatgaccca |
| *OsCRD1* | cggactataaccagaccca | tggccttccagtcgttgag |
| *OsCHLG* | ccactggacagttgaagat | atttacaatagcaatccct |
| *OsCHLP* | ttctacggctgggtgttcc | gtagctgtcgaaggtcatc |
