# Supplementary material for: Hybrid weakness in a rice interspecific hybrid is nitrogen-dependent, and accompanied by changes in gene expression at both total transcript level and parental allele partitioning
Source: PLoS One. 2017 Mar 1;12(3):e0172919. doi: 10.1371/journal.pone.0172919 (PMC5332110; doi:10.1371/journal.pone.0172919)
Supplement: S4 Table — Note: a, primers used in real-time qRT-PCR; b, primers used in pyrosequencing; bio, biotinylated primer. (DOCX) [file pone.0172919.s006.docx]

**S4 Table Primers used in real-time qRT-PCR and pyrosequencing**

| **Gene name** | **Forward primer (5'-3')** | **Reverse primer (5'-3')** | **Pyroseq primer (5'-3')** |
| --- | --- | --- | --- |
| *OsGS1;1^a^* | tgtcagcaccgataccacagt | tacccacaggctatcttcaagg |  |
| *OsGS1;2^a^* | gcatctacgccgggatcaa | ccagccaagaggccagtt |  |
| *OsGS2^ab^* | aacgtaacagggctgcaca | ccagccaagaggccagtt ^bio^ | actttgctccagagagac |
| *OsFd-GOGAT^a^* | gtatcagtggtaggtcgctatgca | tcgaagcatccccttgtagact |  |
| *OsGDH1^a^* | atatgggaaccaactctcagacaa | tagcaaacagaactccccttcc |  |
| *OsGDH2 ^ab^* | gggaagccaatagatcttggt | catgttcagccaaaagagcc ^bio^ | ccaatagatcttggtgg |
| *OsPSBA^a^* | gggatttggttcactgctttag | attaataacgcgacctttggcta |  |
| *OsPSAB ^ab^* | ctccagaaaagcaaatcttgatc | caaccatccgggtaaccata ^bio^ | ggctcatggtaagacg |
| *OsATPD1^a^* | ttcctgtcggtggagctact | gcggatctatgaatagggaatgt |  |
| *OsAtTPD2^a^* | gcgtggaggaaaaatcggactat | cccctactccgccaaatacg |  |
| *OsHEMA ^ab^* | tgaagaggctgctgttcttagtac^bio^ | cacggttccacgataaagctac | ggttccacgataaagctac |
| *OsHEML^a^* | cccggttttctgaatgctctc | accataagcgccaactggaag |  |
| *OsHEME1 ^ab^* | taccaagtaaactctggggctca | caagtggtacattggggcatt ^bio^ | tcaagcagattgtgaataa |
| *OsCHLI ^ab^* | agagagagctcggttcgacag^bio^ | gcttgtcttgttcctccaagtag | tgtcttgttcctccaagta |
| *OsHEMY^a^* | tgaggagtcggtggaggactt | agctttgaaggatcaccagcatac |  |
| *OsPORA^a^* | tgaaccacctgggccact | atggagccgaggatgatga |  |
| *OsPORB ^ab^* | cgggaacacgaacacgct^bio^ | catggcggagctcgacac | cggagctcgacacgc |
| *OsPAO ^ab^* | tttgctcaccacaaggtcact | gaccctgaatatccccatgc ^bio^ | tcaagatggaatcaagtg |
| *OsCRD1^a^* | ggcacctcaaggcgaacc | tggccttccagtcgttga |  |
| *OsCHLG^a^* | tcctgatattgttgtcctgacttc | atttacaatagcaatccctagcc |  |
| *OsCHLP^a^* | cggatggtggaggagagc | cgtcgagcaccttgtacgt |  |
| *OsUBQ5* | accacttcgaccgccactact | acgcctaagcctgctggtt |  |
